# Expression of recombination antimicrobial protein PIL22-PBD-2 in *Pichia pastoris* and verification of its biological function in vitro

**DOI:** 10.1186/s13567-024-01428-1

**Published:** 2025-03-07

**Authors:** Xian Li, Pengfei Qiu, Menglong Yue, Ying Zhang, Congshang Lei, Jingyu Wang, Xiwen Chen, Xuefeng Qi

**Affiliations:** 1 College of Veterinary Medicine, Northwest A&F University, Yangling, Shaanxi China; 2 Key Laboratory of Ruminant Disease Prevention and Control (West), Ministry of Agriculture and Rural Affairs, Yangling, China; 3 Animal Disease Prevention and Control & Healthy Breeding Engineering Technology Research Center, Mianyang Normal University, Mianyang, Sichuan China

**Keywords:** Antibiotic-resistant, recombinant antimicrobial protein, eukaryotic expression, intestinal repair, feed additive

## Abstract

Finding suitable alternatives to antibiotics as feed additives is challenging for the livestock industry. Porcine beta-defensin 2 (PBD-2) is an endogenous antimicrobial peptide produced by pigs. Due to its broad-spectrum antibacterial activity against various microorganisms and its low tendency for drug resistance, it is considered a potential substitute for antibiotics. Additionally, given its strong ability to repair intestinal epithelial damage and maintain intestinal mucosal barrier function, porcine interleukin-22 (PIL-22) is a potential feed additive to combat intestinal damage caused by intestinal pathogens in piglets. In this study, the amino acid sequences of PBD-2 and PIL-22 were combined to express the fusion protein in *Pichia pastoris*, and its biological activity was evaluated in vitro. Our results showed that the PIL22-PBD-2 exhibits broad-spectrum antibacterial activity against multidrug-resistant enterotoxigenic *Escherichia coli* O8 (ETEC O8), *Escherichia coli* (*E. coli*), *Salmonella typhimurium,* and *Staphylococcus aureus* (*S. aureus*). PIL22-PBD-2 demonstrated wound repair capability through a healing assay in the intestinal porcine epithelial cell line-J2 (IPEC-J2). Furthermore, PIL22-PBD-2 significantly enhanced the expression of the major intercellular junction-associated proteins ZO-1 and E-cadherin in IPEC-J2. It is important to note that PIL22-PBD-2 reduced intestinal epithelial cell apoptosis (*p* < 0.05) considerably and decreased bacterial adhesion (*p* < 0.05) in ETEC O8-challenged IPEC-J2. We also found that the PIL22-PBD-2 treatment attenuated ETEC O8-induced inflammatory responses in IPEC-J2 by exerting antibacterial activity, increasing the expression of endogenous antimicrobial peptides, and significantly decreasing the mRNA expression levels of IL-6 and TNF-α (*p* < 0.05). In conclusion, our studies demonstrate that PIL22-PBD-2 has a positive effect on inhibiting pathogenic bacteria and repairing intestinal damage.

**Supplementary Information:**

The online version contains supplementary material available at 10.1186/s13567-024-01428-1.

## Introduction

It is widely acknowledged that antibiotics have a positive impact on animal husbandry development because of their antibacterial, antiviral, and growth-promoting effects [1, 2]. However, long-term use of large quantities of antibiotics can cause increased bacterial resistance and residual effects during treatment [3]. Bacterial resistance caused by antibiotic abuse has become one of the biggest threats to the safety and development of husbandry. As a result of this resistance, there has been an increase in antibiotic-sensitive bacteria that no longer respond to antibiotics [4, 5].

Developing broad-spectrum and highly effective antibiotic alternatives against intestinal bacterial infections is crucial for several reasons. For example, enterotoxigenic *Escherichia coli* (ETEC), which causes post-weaning diarrhoea (PWD) in piglets, has a detrimental impact on animal health and the economy of pig production [6]. Furthermore, bacterial toxins destroy tight junction integrity and intestinal epithelium apoptosis, disrupting intestinal homeostasis and damaging intestinal barrier functions [7]. The intestinal epithelium barrier not only contributes to the absorption of nutrients but also helps prevent pathogens and toxins from entering circulation from the intestinal lumen [7, 8]. Multiple pathogens cause damage to the intestinal epithelial barrier, which subsequently activates the innate immune response and produces large numbers of pro-inflammatory cytokines such as interleukin-6 (IL-6) and tumour necrosis factor-α (TNF-α) [7, 9].

Nonconventional antimicrobials, such as phages [10], plant-derived compounds [11], probiotics [12], and antimicrobial peptides (AMPs) [13], are currently being explored as pharmacologically significant alternatives to combat the menace of antimicrobial resistance. Studies have shown that AMPs have a broad spectrum of activities against bacteria, fungi, and viruses [14]. They can cause cell death by forming ion channels in cell membranes [15] or can enter cells to act on nucleic acids and affect the synthesis and replication of DNA and RNA [16–18]. AMPs not only have the ability to simultaneously target multiple drugs, making them less susceptible to resistance, but they are also a part of the innate immune system that plays a role in immunomodulation in all species [19–22]. In addition to their well-recognised direct antimicrobial activities, defensins, a major group of AMPs found in many mammalian species, can stimulate diverse biological effects in initiating and amplifying host innate and adaptive immune responses. Porcine beta-defensin 2 (PBD-2) is widely expressed in pig organs, exhibiting a broad spectrum of bactericidal activities. It is also established that PBD-2 modulates the effect of innate immunity [23–25]. Furthermore, PBD-2 can be used as a medicinal feed additive for weaned piglets as it has been shown to promote piglet growth, reduce piglet diarrhoea, improve growth performance and caecal microbiota, and decrease the expression of inflammatory cytokines [26–28].

Further to the AMPs produced by the body to maintain its resistance, some cytokines can induce the body to produce AMPs to enhance resistance to pathogens. Interleukin-22 (IL-22), recognised as a cytokine that protects epithelium, has also been widely studied for its various effects on cell proliferation, tissue regeneration, cellular defence, and inflammation [29]. Previous studies have shown that IL-22 in the intestine can stimulate intestinal epithelial cells to produce AMPs, enhance the intestinal epithelial mucus barrier, and promote intestinal repair [30–32]. A further study provided a theoretical basis for curing intestinal inflammation caused by ETEC K88 infection and epithelial apoptosis induced by deoxynivalenol using IL-22 in pigs [8].

Until now, few studies have investigated the functions of IL-22 fusion expression alongside AMPs. However, many studies have demonstrated that the fusion of AMPs exhibits higher antibacterial activity and lower cytotoxicity than single antimicrobial peptides [33–36]. Moreover, the fusion of AMPs and cytokines can enhance the antibacterial defence response in mice [37]. In the current study, we synthesised and expressed the recombinant antimicrobial protein PIL22-PBD-2 with the help of *Pichia pastoris* (*P. pastoris*) and detected the effect of PIL22-PBD-2 on inhibiting pathogenic bacteria and repairing intestinal damage in vitro. Our data will provide a useful reference for further studies on AMPs as clinical alternative antibiotic agents.

## Materials and methods

### Protein, vectors, bacterial strain, and cell line

The proteins PBD-2 (Additional files 1A, B and C) and porcine IL-22 (PIL-22; Additional files 1D, E and F) were expressed in our laboratory using the *P. pastor*is strain GS115 (Invitrogen, USA). The *P. pastoris* GS115 and expression vectors pPIC9K (p9K; Invitrogen, USA) were routinely available in our laboratory.

The ETEC O8 strain, resistant to kanamycin, ampicillin, streptomycin and ofloxacin (specific gene: *orf469*; GenBank ID: AB010150; Primer sequence 5′-3′: F-CCAGAGGCATAATCAGAAATAACAG, R-GCAGAGTTAGTCAACAAA-AGGTCAG [38];) was isolated from the faeces of clinically diarrheal piglets and maintained in our laboratory (Additional file 2).

The bacterial strains *Escherichia coli* (*E. coli*) ATCC25922, *Staphylococcus aureus* (*S. aureus*) ATCC25923, and *Salmonella typhimurium* ATCC19659 were provided by the College of Veterinary Medicine at Northwest A&F University and stored at −80 ℃ in nutrient broth (NB).

All strains were cultured in NB at 37 ℃ with agitation at 150 rpm. The intestinal porcine epithelial cell line-J2 (IPEC-J2) was obtained from the China Center for Type Culture Collection in Wuhan and is maintained in liquid nitrogen with a mixture of 90% dimethyl sulfoxide (DMSO) and 10% foetal bovine serum (FBS, PAN, Germany). The IPEC-J2 was cultured in Dulbecco’s Modified Eagles Media (DMEM)/high glucose (containing 8 mmol/L of L-glutamine and 4.5 g/L of glucose; Hyclone, USA) supplemented with 10% FBS (PAN, Germany), 100 IU/mL penicillin and 100 μg/mL streptomycin (Gibco, CA, USA) at 37 ℃ in a 5% CO_2_ atmosphere.

### Construction of recombinant plasmid pPIC9K-PIL22-PBD-2

The plasmid pPIC9K-PIL22-PBD-2 was constructed by linking the porcine IL-22 gene (GenBank: KX588234.1) and the porcine beta-defensin 2 gene (GenBank: KF671230.1) using the spacer sequence (GGGGSGGGGS). Subsequently, the recombinant sequence was connected to the 6-histidine tag sequence by the spacer sequence (GGGGS), and the resultant construct was named PIL22-PBD-2. According to the codon usage of *P. pastoris*, the GC content of the PIL22-PBD-2 DNA sequence was optimised using the GenScript GenSmart codon optimisation tool, increasing the average GC content from 39 to 47%.

The plasmid pUC57-PIL22-PBD-2 was synthesised by Nanjing GenScript Biotech Co., Ltd (China) (Table 1 and Additional file 3). The PIL22-PBD-2 and pPIC9K fragments were obtained through double enzyme digestion using *Eco*R I (Takara Biotechnology, Dalian, China) and *No*t I (Takara Biotechnology, Dalian, China) on the plasmids pUC57-PIL22-PBD-2 and pPIC9K, respectively. The obtained PIL22-PBD2 fragment was ligated with the enzyme-digested pPIC9K vector using T4 DNA ligase (Takara Biotechnology, Dalian, China) at 16 ℃ for 8 h in a molar ratio of 7:1. The connecting product (pPIC9K-PIL22-PBD2) was transformed into *E. coli* TOP10 chemically competent cells (Tsingke Biotechnology, Beijing, China) by heat shock. The transformed cells were then coated on Luria Bertani (LB) agar plates containing ampicillin antibiotics (50 µg/mL). *E. coli* TOP10 cells containing the plasmids pPIC9K-PIL22-PBD2 were cultivated in LB medium supplemented with 50 µg/mL ampicillin for 16 h at 37 ℃ with shaking at 180 rpm. These plasmids were purified using an Endofree plasmid kit (OMEGA, America). A NanoDrop microvolume spectrophotometer (NanoDrop 2000, Thermo Scientific, Waltham, MA, USA) was used for concentration determination. After confirming the correct construction of the plasmids, they were stored at −20 ℃ until use.

**Table 1:** **The amino acid sequences of PIL-22 and PBD-2**.

Name	Sequences	Origin of Sequences
PIL-22	PITHHCKLDQSNFQQPYITNRTFTLAQEASLADNNTDVRLIGNNLFQGVNQMRERCYLVKQVLNFTLEEVLFPNSDRFHPYMQEVASFLDSLSKKLSQCRIKGDDQHIQRNVNNFKDIVKKLGESGEIKVIGELYLLFMALKNECTLPGHSWKMDN	KX588234.1
PBD-2	RALCLLLLTVCLLSSQLAAGINLLTGLGQRSDHYICAKKGGTCNFSPCPLFNRIEGTCYSGKAKCCIR	KF671230.1
6-histidine	HHHHHH	
spacer sequence	(GGGGS)n	[39]

### Transformation and colony PCR assay

Before electrotransformation, the plasmid construct p9K-PIL22-PBD-2 was linearised at the *Sal* I site. After mixing 10 μL of linearised recombinant plasmids with 100 μL of competent *P. pastoris* GS115 cells, yeast cells were immediately transferred to a pre-chilled electroporation cuvette and incubated on ice for 15 min. To perform electrotransformation (Bio-Rad, USA), a charging voltage of 1.5 kV was used with the capacitance set at 25 μF. Resistance was maintained at 200 Ω. Transformants were inoculated on minimal dextrose (MD) medium plates (1.34% YNB, 0.00004% biotin, 2% dextrose, 1.5% agar) without histidine. Clones from the MD plates were then selected on YPD/geneticin (G418; Solarbio, China) plates (1% yeast extract, 2% peptone, 2% dextrose, 2% agar, and 4 mg/mL G418).

Colony PCR was performed to verify the integration of plasmid p9K-PIL22-PBD2 into the genome of *P. pastoris* GS115 grown on YPD plates containing G418. Briefly, colonies that grew on YPD plates were expanded in liquid YPD medium, with their genomic DNA isolated using the yeast DNA kit (Solarbio, Beijing, China). The primers used for PCR are listed in Table 2. The integration of the p9K-PIL22-PBD2 plasmid construct was verified by PCR using 300 ng of genomic DNA as a template. PCR conditions were as follows: pre-denaturation stage at 95 ℃ for 5 min, followed by 34 cycles of denaturation at 95 ℃ for 30 s, annealing at 58 ℃ for 30 s, and extension at 72 ℃ for 50 s, with a final extension at 72 ℃ for 10 min. For amplification controls, 100 ng of empty plasmids p9K were used as a negative control.

**Table 2:** **Primer sequences for** ***P. pastoris*** **GS115, PBD-2, PIL-22, and PIL22-PBD-2**

Gene name	Primer sequences	Length (bp)
5′/3′ AOX1	F: GACTGGTTCCAATTGACAAGC R: GCAAATGGCATTCTGACATCC	481
PBD-2	F: ATGAGAGCTTTGTGTTTGTTG R: TTAATGGTGATGATGATGATG	243
PIL-22	F: CCAATTACCCACCACTGT R: TTAATGGTGATGATGATGATG	522
PIL22-PBD-2	F: ATGCCCATTACTCATCATTG R: TCAGTGGTGATGATGGTGGTG	741

### Yeast expression of PIL22-PBD-2

In line with previous methodology, the yeast expression system was used to express PIL22-PBD-2 [40]. Recombinants bearing the target gene were inoculated into 10 mL of buffered glycerol-complex medium (BMGY; 1% yeast extract, 2% peptone, 1.34% YNB, 0.00004% biotin, 1% glycerol, 100 mM potassium phosphate, pH 6.0) and incubated at 28 ℃ for 2 days. The cells in each culture were collected by centrifugation at 4000 × *g* for 5 min and then individually inoculated into 100 mL of buffered minimal methanol YP medium (BMMY), which is the same as BMGY but with glycerol replaced by 1% methanol. A total of 2% (v/v) methanol was added every 24 h to induce protein expression. The p9K was also operated according to the above experimental procedure. Additionally, PIL22-PBD-2 in the supernatant was separated and purified using the His-tag Protein Purification Kit (7sea Biotech, China). The Bradford method (Beyotime, China) established protein concentrations and determinations. The specific protein production was confirmed by Western blotting with a mouse anti-HIS tag monoclonal antibody (1:5000 dilution; Abbkine Scientific Co., Ltd., Wuhan, China) and a horseradish peroxidase (HRP)-conjugated goat anti-mouse IgG antibody (1:5000 dilution; Abbkine Scientific Co., Ltd., Wuhan, China).

### Antibacterial assay

The bacteriostatic activity of recombinant PIL22-PBD-2 protein against four kinds of bacteria (ETEC O8, *E. coli* ATCC25922, *Salmonella* ATCC19659, and *S. aureus* ATCC25923) was detected using the Oxford Cup bacteriostatic test. Briefly, the bacterial strains were incubated at 37 ℃ for 12 h on a NB medium. Subsequently, two hundred microliters of suspension containing 10^6^ CFU/mL of bacteria were spread on a nutrient agar medium. Oxford cups (6 mm diameter) were placed on the inoculated agar. A micropipette was used to add 100 μL of PIL22-PBD-2 protein, kanamycin, and sterile water. The recombinant PIL22-PBD-2 protein and kanamycin concentration was 100 μg/mL, with sterile water as the negative control. The plates were moved to the incubator and cultured at 37 ℃ for 16 to 18 h.

### Minimum inhibitory concentration (MIC) assay

The previously described method determined the MIC [7]. Briefly, bacteria cultured overnight were diluted to 10^5^ CFU/mL and seeded in 96-well plates at 100 μL per well. The recombinant protein was continuously diluted from a 256 μg/mL concentration by a factor of 2 (100 μL per well). The same volume of sterile medium and the same concentration of bacterial solution were used as negative and positive controls, respectively. The MIC was detected at OD_600_ nm after the mixed system was incubated at 37 ℃ for 24 h. Each experiment was repeated thrice.

### Transmission electron microscopy (TEM)

To display the bactericidal effect of PIL22-PBD-2 on bacteria, TEM was used to observe the morphological changes. Bacteria (1.0 × 10^6^ CFU/mL) were incubated with MIC and 0.5 MIC of PIL22-PBD-2 at 37 ℃ for 2 h, with bacteria added to the sterile PBS as the controls. The cultures were centrifuged at 2000 × *g* for 5 min, and the pellet was fixed with 0.5% glutaraldehyde for 5 min at room temperature. The fixed samples were centrifuged at 2000 × *g* for 5 min, and the pellet was resuspended in the fixing solution before being stored at 4 ℃.

The bacteria were subsequently postfixed with osmium tetroxide (Sigma-Aldrich, Schnelldorf, Germany), dehydrated with acetone (Panreac, Barcelona, Spain), embedded in resin, and sectioned with an ultramicrotome (Ultramicrotome UC7, Leica, Viena, Austria). Ultrathin Sects. (50–70 nm) were stained with 2% uranyl acetate (Thermo Fisher Scientific, Waltham, MA, USA) for 10 min, a lead staining solution for 5 min, and finally analysed on a JEOL 1010 transmission electron microscope with a CCD Orius digital camera (Gatan). The damage rate of bacteria in TEM images was analysed.

Bacteria with intact surfaces and dense internal structures were judged to be undamaged. Conversely, the presence of holes, gaps, cavitation, unclear boundaries, and morphological abnormalities in bacteria was considered as damage. The three visual fields of the treated bacteria were randomly selected to calculate their damage rates. The obtained data was statistically analysed using GraphPad Prism 8. (Bacterial damage rate = number of damaged bacteria in the visual field/total number of bacteria in the visual field).

### Cell proliferation assay and wound healing assay

The CCK8 assay (Neobioscience) was used to evaluate cell proliferation after treatment with different concentrations of PBD-2, PIL-22, and PIL22-PBD-2. Briefly, IPEC-J2 cells were seeded into 96-well plates at a density of 1 × 10^4^ cells and incubated in a 5% CO_2_ incubator at 37 ℃ for 12 h. PBD-2, PIL-22, and PIL22-PBD-2 were diluted to concentrations of 128, 64, 32, 16, 8, 4, and 2 μg/mL (100 μL/well) in DMEM. Control group wells were simultaneously set up. Each group had six replicates, with the cells cultured in the cell incubator for 24 h. Lastly, 10 μL of CCK8 was added to each well, and the culture was incubated for 1 h. The OD_450_ nm value was measured using a microplate reader, and the cell survival rate was calculated.

Cell viability = [(As-Ab)/(Ac-Ab)] × 100%

As: Absorbance of experimental wells (including cells, culture medium, CCK8 solution and drug solution)

Ac: Absorbance of control well (including cells, culture medium and CCK8 solution)

Ab: Absorbance of blank wells (including cells and culture medium)

Wound healing experiments were conducted as previously described to evaluate further the proliferation of recombinant proteins on IPEC-J2 [41]. Briefly, IPEC-J2 cells were seeded into a 6-well plate at a cell density of approximately 1 × 10^6^ cells/well. The plate was then incubated at 37 ℃ with 5% CO_2_ until the confluence rate reached 80%. The cells were scratched by a pipette tip and washed gently with Hanks’ Balanced Salt solution (HBSS) (Hyclone, USA). Next, PBD-2, PIL-22, and PIL22-PBD-2 diluted with DMEM medium were added to each well with final concentrations of 4 and 1 μg/mL. Finally, the plate was incubated at 37 ℃ for 12 h in 5% CO_2_. The wound healing was observed under the microscope at 0 h, 6 h, and 12 h (Zeiss, Germany). Subsequently, the wound areas were measured at the same magnification using ImageJ software.

### qPCR and Western blot analysis of proteins at intercellular junctions

Both qPCR and Western blot were used to detect the intercellular junction proteins ZO-1 and E-cadherin expression. IPEC-J2 cells were treated with PBD-2, PIL-22, and PIL22-PBD-2 for 24 h, then harvested. The total RNA was extracted using Trizol (Accurate Biology, China) following the manufacturer’s instructions. Cells treated with PBD-2, PIL-22, and PIL22-PBD-2 were lysed with 0.5 mL of RIPA buffer (Solarbio, China) containing 10 mM phenylmethanesulfonyl fluoride (PMSF; Solarbio, China). The supernatant of lysed cells was obtained by centrifugation at 4 ℃, 10,000 × *g* for 5 min, followed by protein quantitation using a BCA protein assay kit (Beyotime, China). Cell lysates containing equal amounts of protein were then separated by SDS-PAGE and transferred to a PVDF membrane (Millipore, USA). Membranes were blocked with 5% skim milk powder for 2 h at room temperature. The blotted membranes were probed with primary antibodies at 4 ℃ overnight. These antibodies included mouse anti-pig ZO-1 monoclonal antibody (Proteintech, China; 1:5000), rabbit-derived E-cadherin polyclonal antibody (Immunoway, USA; 1:3000), and mouse anti-β-actin antibody (Yeasen, China; 1:5000). The membranes were then washed and incubated with goat anti-rabbit or goat anti-mouse IgG-HRP (Yeasen, China; 1:5000) for 2 h at room temperature. Membranes were washed four times in PBST buffer (5 min per wash) (Solarbio, China). The target protein was visualised using the enhanced chemiluminescence (ECL) system (Bio-Rad, USA) and analysed with ImageJ software.

### Immunofluorescence assay (IFA)

The intercellular junction proteins were detected using the immunofluorescence method. Briefly, IPEC-J2 monolayers were inoculated with PBD-2, PIL-22, and PIL22-PBD-2 at concentrations of 4 and 1 μg/mL. After 24 h, the cells were fixed and then incubated with mouse anti-pig ZO-1 monoclonal antibody (1:500) and rabbit-derived E-cadherin polyclonal antibody (1:300). Following this, the cells were incubated with FITC-conjugated goat anti-mouse or TRITC-conjugated goat anti-rabbit IgG (Yeasen, China; 1:200). Fluorescence was visualised using confocal laser microscopy (Nikon, Japan). Next, ImageJ software was used to analyse the relative fluorescence intensity.

### Assessment of apoptosis by flow cytometry

IPEC-J2 cells were pre-treated with PBD-2, PIL-22, and PIL22-PBD-2 at concentrations of 1 or 4 μg/mL for 12 h, followed by a 4 h exposure to ETEC O8 (MOI = 10) without antibiotics, to evaluate the protective effect of proteins against ETEC O8. The control groups consisted of cells treated either with the proteins for 16 h or without the proteins for 12 h, then exposed to ETEC O8 for 4 h.

The treated cells were washed thrice with PBS to remove residual pancreatic enzymes. Afterwards, ETEC O8-stimulated apoptosis in IPEC-J2 was determined using the PI (propidium iodide) and annexin V-FITC staining method (Beyotime, China)*.* The apoptosis rate was determined using flow cytometry (FACSCantoII, BD Biosciences, USA), and all flow cytometric data were analysed using FlowJo software (BD Biosciences, USA). After treating IPEC-J2 cells with PIL-22 and PIL22-PBD-2 at concentrations of 4 μg/mL, groups treated with or without ETEC O8 were collected for later examination to detect the levels of inflammatory factors and endogenous defensins mRNA expression.

### Adhesion of ETEC O8

IPEC-J2 cells were cultured in six-well tissue-culture plates until they reached confluency. The cells were then washed thrice with antibiotic-free medium before being treated with 1 mL/well of DMEM medium (without antibiotics) that contained PBD-2, PIL-22, and PIL22-PBD-2 at concentrations of either 1 or 4 μg/mL for 12 h. The cells were then challenged with ETEC O8 (MOI = 10) for another 4 h (without antibiotics). PBS was used as a control group for 12 h, followed by ETEC O8 challenge (MOI = 10) for another 4 h. The cells in the six-well plate were incubated with 0.5% Triton X-100 in PBS at room temperature for 5 min, allowing the bacteria to be released. After continuous tenfold dilution with sterile saline, 100 μL were plated on LB solid agar medium and incubated at 37 ℃ for 12 h. Each group was repeated thrice to ensure accuracy. The number of adhering bacteria was determined using colony-counting methods.

### qRT-PCR

IPEC-J2 cells were harvested, and the total RNA was extracted using Trizol (Accurate Biology, China) according to the manufacturer’s instructions. The cDNA was synthesised using Trans Script^®^ First-Strand cDNA Synthesis Super Mix (Trans Gen Biotech, China). The cDNA in 20 μL Trans Start^®^ Top Green qPCR Super Mix (Trans Gen Biotech, China) was amplified by qRT-PCR using the Light Cycler 96 real-time PCR system (Bio-Rad, USA) with designed primers (Table 3). The reaction procedure included a pre-denaturation stage (95 ℃, 5 min), a 40 cycles PCR stage (denaturation in 95 ℃ for 15 s, annealing in 60 ℃ for 30 s, extension in 72 ℃ for 30 s). The melting curve was obtained by gradually warming from 65 ℃ to 95 ℃. Levels of mRNA were calculated using the 2^−ΔΔCt^ method and normalised to those of β-actin mRNA.

**Table 3:** **Primers for 2**^**−ΔΔCt**^** method qRT-PCR in this study**.

Gene name	Primer sequences	Origin of Sequences
ZO-1	F: CCCCAGGGACTGGAAGAAATCAC R: CCCCTCCACTTCCTCTAGCCAAG	[41]
E-cadherin	F: TGAGAAATGAGTGTGCTTTTGTGCC R: TGGCCAGACTAAGACATGAACCTC	[41]
PBD-1	F: CTCCTCCTTGTATTCCTCCT R: GGTGCCGATCTGTTTCAT	[42]
PBD-2	F: GACTGTCTGCCTCCTCTC R: GGTCCCTTCAATCTGTTG	[42]
IL-6	F: CCTCGGCAAAATCTCTGCAA R: TGAAACTCCACAAGACCGGT	[43]
TNF-α	F: GCCCTTCCACCAACGTTTTC R: TCCCAGGTAGATGGGTTCGT	[43]
β-actin	F: AGATCAAGATCATCGCGCCT R: ATGCAACTAACAGTCCGCCT	[43]

### Statistical analysis

All values should be expressed as the arithmetic mean of triplicates ± standard error of the mean (SEM). Two-way ANOVA determined significance with a Bonferroni’s post-test or paired Student’s *t*-test. Values of *p* < 0.05 were considered to indicate statistical significance. The statistics software GraphPad Prism 8 (GraphPad Software, Inc., San Diego, CA, USA) was used.

## Results

### Expression of recombinant PIL22-PBD-2

**Figure 1:**
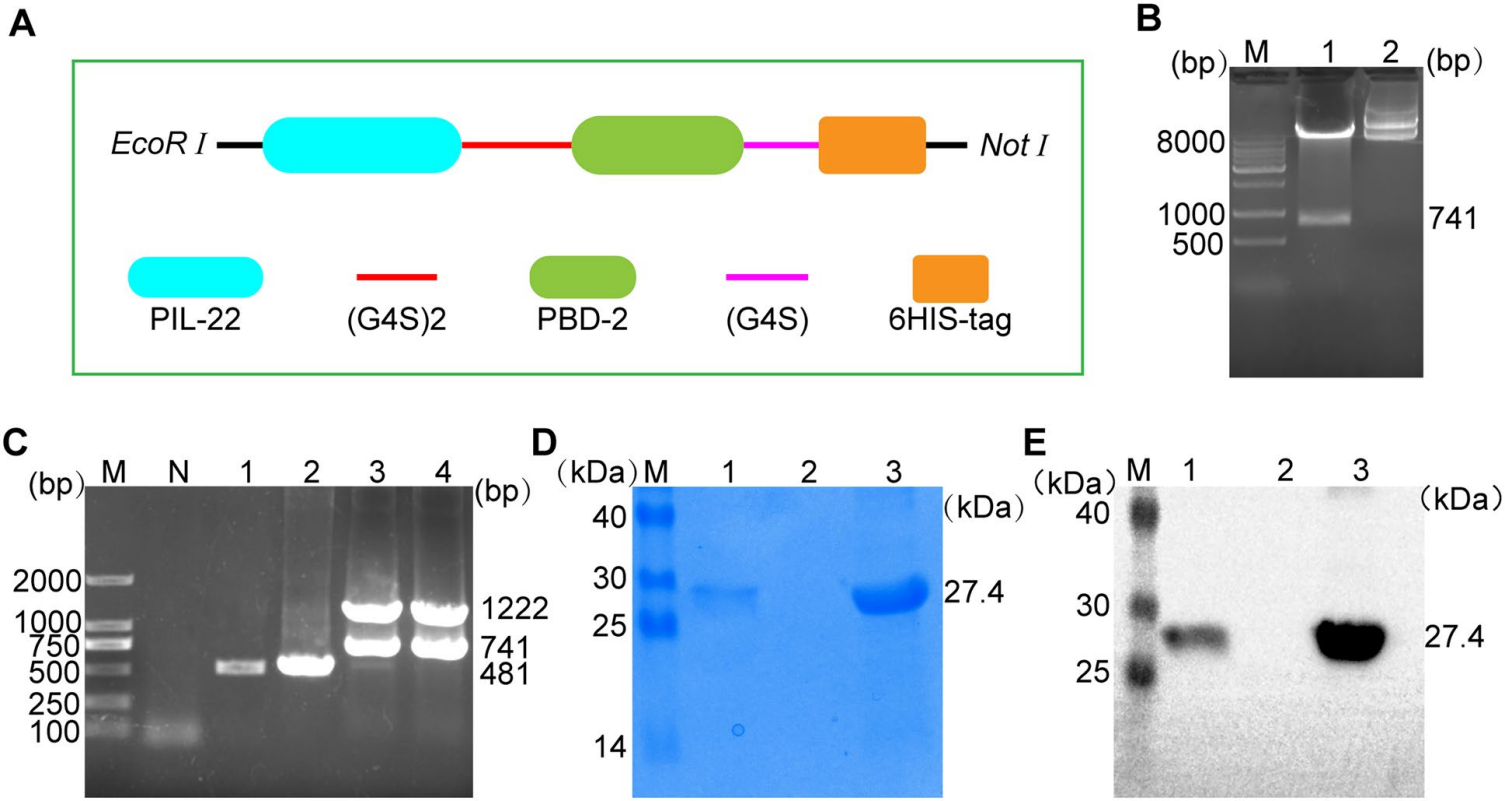
**Construction and expression of recombinant PIL22-PBD-2 protein in** ***P. pastoris***. **A** Schematic diagram of recombinant proteins design. **B** Identification of the plasmids pPIC9k-PIL22-PBD-2 by double enzyme digestion. M: DL10000 DNA marker; Lane 1: plasmid p9k-PIL22-PBD-2 digested with *Eco*R I-*No*t I; Lane 2: p9k-PIL22-PBD-2 without *Eco*R I-*No*t I. **C** Colony PCR identification of recombinant *P. pastoris* containing PIL22-PBD-2 gene. Lane M: DL2000 DNA marker; Lane N: negative control; Lane 1 and 2: *P. pastoris* GS115; Lane 3 and 4: *P. pastoris* GS115 containing PIL22-PBD-2 gene; SDS-PAGE (**D**) and WB (**E**) analyses of PIL22-PBD-2 protein. Lane M: protein marker. Lane 1: the fermentation supernatant of PIL22-PBD-2. Lane 2: the fermentation supernatant of blank vector as negative control; Lane 3: purified PIL22-PBD-2 protein.

The yeast expression plasmid p9K-PIL22-PBD-2 was successfully constructed. Enzyme digestion was performed with *Eco*R I and *No*t I, yielding a fragment of 741 bp (Figures 1A and B) to verify the recombinant plasmid. The recombinant plasmid was electrotransformed into the GS115 AOX1 gene and verified by colony PCR. As shown in Figure 1C, a 1222 bp fragment and a 741 bp fragment were amplified with *P. pastoris* AOX1 primers and PIL22-PBD-2 gene primers, respectively. After continuous induction with 2% (v/v) methanol for 96 h, PIL22-PBD-2 was detected in the supernatant of the fermentation broth using SDS-PAGE (Figure 1D). Furthermore, using a mouse anti-HIS tag monoclonal antibody (Figure 1E), the Western blot confirmed the band of the purified sample as PIL22-PBD-2. Cumulatively, these results indicate that the recombinant plasmid was correctly constructed and that the *P. pastoris* GS115 strain successfully expressed PIL22-PBD-2.

### In vitro antibacterial activity of PIL22-PBD-2

**Figure 2:**
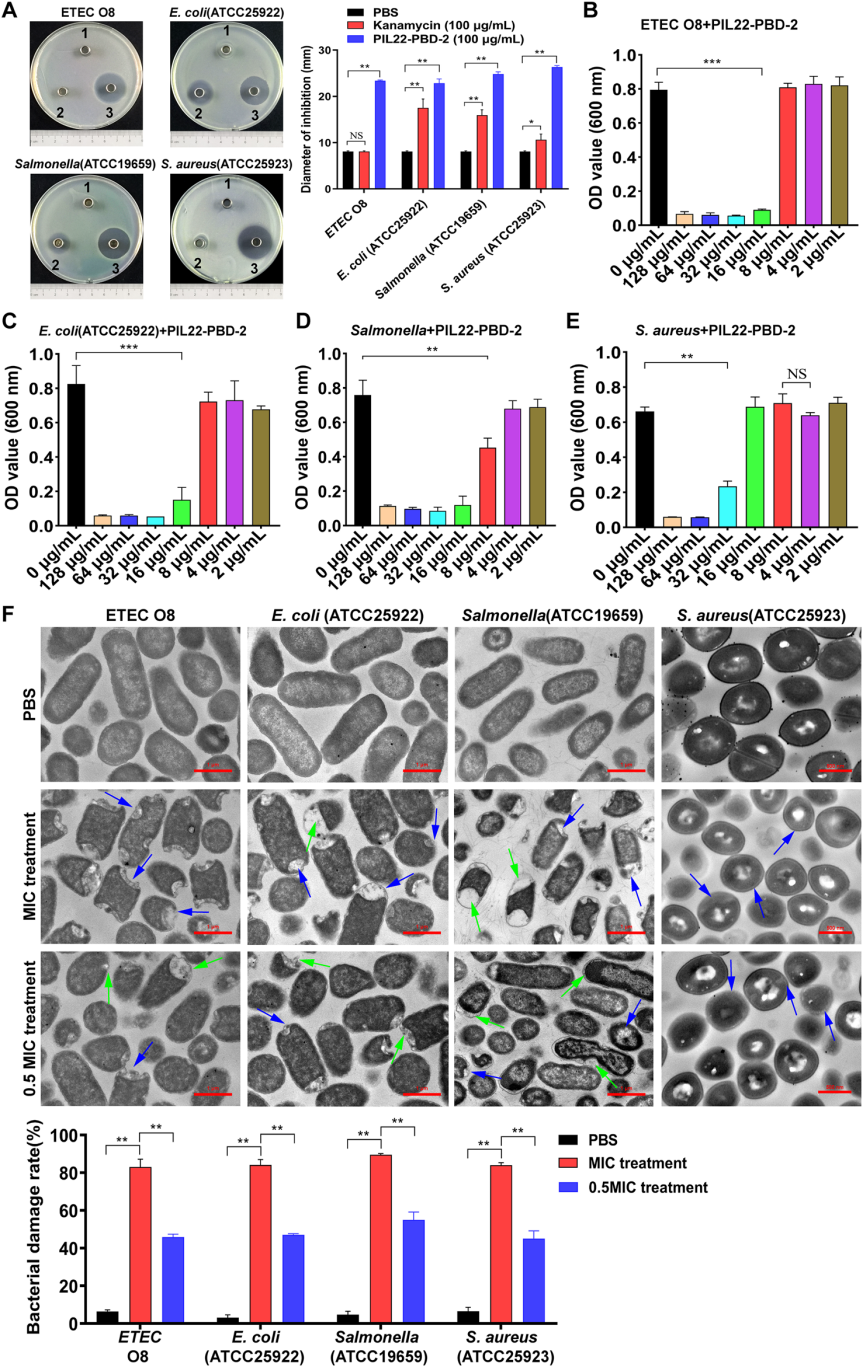
**The Antibacterial effect of the PIL22-PBD-2**. **A** Representative bacteriostasis plates of sterile water (1), kanamycin (100 μg/mL) (2), and PIL22-PBD-2 (100 μg/mL) (3) were tested for their effects on ETEC O8, *E. coli* ATCC25922, *Salmonella typhimurium* ATCC19659, and *S. aureus* ATCC25923. MIC of PIL22-PBD-2 on ETEC O8 (**B**), *E. coli* ATCC25922 (**C**), *Salmonella typhimurium* ATCC19659 (**D**) and *S. aureus* ATCC25923 (**E**) was determined. Each test was performed in duplicate and repeated three times. **F** The representative field of view for observing the damage caused by PIL22-PBD-2 on pathogenic bacteria was observed under TEM, and the data on bacterial damage rate were statistically analysed. Blue and green arrows are used to indicate bacterial damage. Blue arrows represent holes, blurred borders, and irregular shapes of bacteria, whereas green arrows indicate bacterial cavitation and widened gaps in the cell membrane (wall). *p < 0.05, and **p < 0.01 were compared by *t*-test.

The Oxford Cup bacteriostasis test showed that PIL22-PBD-2 has significant antibacterial activity against *E. coli* (ETEC O8 and ATCC25922), *Salmonella*, and *S. aureus* (Figure 2A). Moreover, the results indicate that MIC is reliable only when the inhibition rate exceeds 90%. The MIC values of PIL22-PBD-2 against ETEC O8, *E. coli* ATCC25922, *Salmonella*, and *S. aureus* were 16 µg/mL (Figure 2B), 16 µg/mL (Figure 2C), 32 µg/mL (Figure 2D), and 64 µg/mL (Figure 2E). Furthermore, morphological and ultrastructural analysis of bacteria treated with 1 or 0.5 MIC concentration of PIL22-PBD-2 for 2 h was conducted using transmission electron microscopy (Figure 2F).

The control group of *E. coli*, *Salmonella*, and *S. aureus* without PIL22-PBD-2 treatment exhibited smooth and intact cell surfaces with dense internal structures. Conversely, compared with the control group, gram-negative bacteria treated with 1 MIC and 0.5 MIC concentrations of PIL22-PBD-2 appeared irregular in shape. Many bright holes and gaps were observed on the damaged cells, leading to their death (Figure 2F: blue arrow). The cell integrity of these bacteria was seriously compromised (*p* < 0.01). The cell walls (membranes) of *Salmonella typhimurium* ATCC19659 showed significant widening of gaps (Figure 2F: green arrow).

Additionally, cytoplasmic leakage and cell cavitation phenomena were observed in the bacteria (Figure 2F: green arrow). When treated in PBS, the *S. aureus* strain ATCC25923 displayed an intact and pristine cell wall configuration. However, upon exposure to different dosages of 1 MIC and 0.5 MIC of PIL22-PBD-2, the microorganism exhibited indistinct membrane borders, reduced cellular dimensions, and abnormal morphologies (Figure 2F: blue arrow). These findings confirm that PIL22-PBD-2 has a broad spectrum of antibacterial properties, indicating its potential as an antibiotic substitute.

### The PIL22-PBD-2 promotes the proliferation and wound healing of IPEC-J2

**Figure 3:**
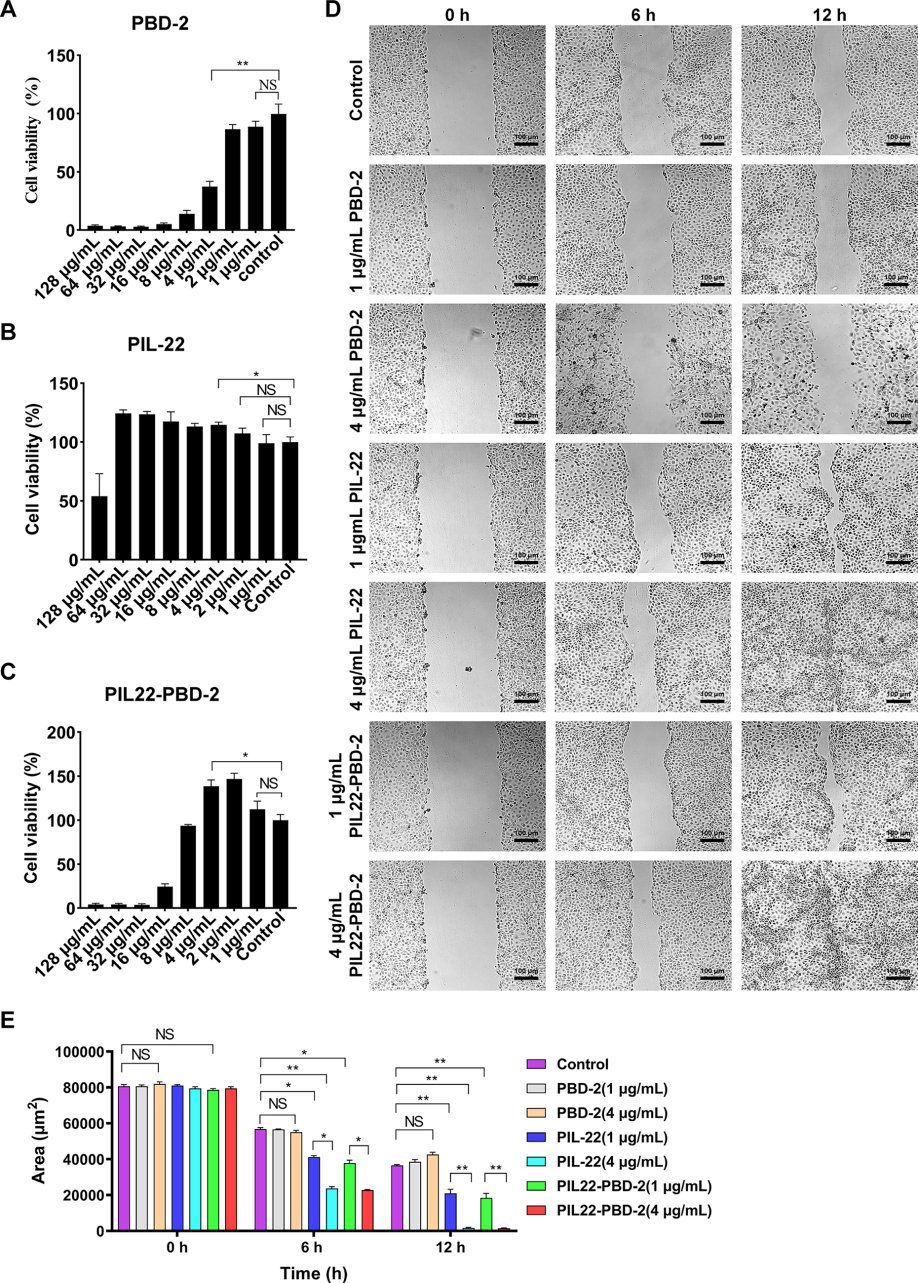
**The PIL22-PBD-2 promotes the proliferation and healing of IPEC-J2 cells**. The effects of PBD-2 (**A**), PIL-22 (**B**) and PIL22-PBD-2 (**C**) on the proliferation of IPEC-J2 cells within 24 h. **D** For wound healing assay, the recombinant proteins of PBD-2, PIL22 and PIL22-PBD-2 treated IPEC-J2 in 12 h. PBS treatment was used as control. **E** Results of statistical analysis of the wound healing areas of IPEC-J treated by PBD-2, PIL22 and PIL22-PBD-2. Error bars indicate standard deviations. *p < 0.05 and **p < 0.01 were compared by two-way ANOVA followed by Bonferroni’s multiple comparisons test.

The working concentrations PBD-2, PIL-22, and PIL22-PBD-2 were determined using a cytotoxicity kit-8 assay to investigate whether they promote the proliferation of IPEC-J2. The results of the CCK8 assay indicated that PBD-2 did not promote the proliferation of IPEC-J2 within the experimental concentration range. In addition, PBD-2 at a concentration of 4 μg/mL caused a decrease in cell viability compared to the control group (Figure 3A).

Interestingly, the final concentration of PIL-22 and PIL22-PBD-2 was 4 μg/mL, which significantly promoted the proliferation of IPEC-J2 after 24 h (*p* < 0.05) (Figures 3B and 3C). To reflect the differences among PBD-2, PIL22, and PIL22-PBD-2, wound healing assays were set up with final concentrations of 4 and 1 μg/mL (Figure 3D). The quantitative analysis of wounded areas showed that PIL-22 and PIL22-PBD-2 substantially promoted the healing of scratches on IPEC-J2 compared to the control group in a concentration-dependent manner (Figure 3E). Furthermore, we found that PBD-2 at a concentration of 4 μg/mL caused partial cell death, while PBD-2 at a concentration of 1 μg/mL did not; however, it also did not significantly promote scratch repair compared to the control group.

### The PIL22-PBD-2 promotes the expression of intercellular junction proteins of IPEC-J2

**Figure 4:**
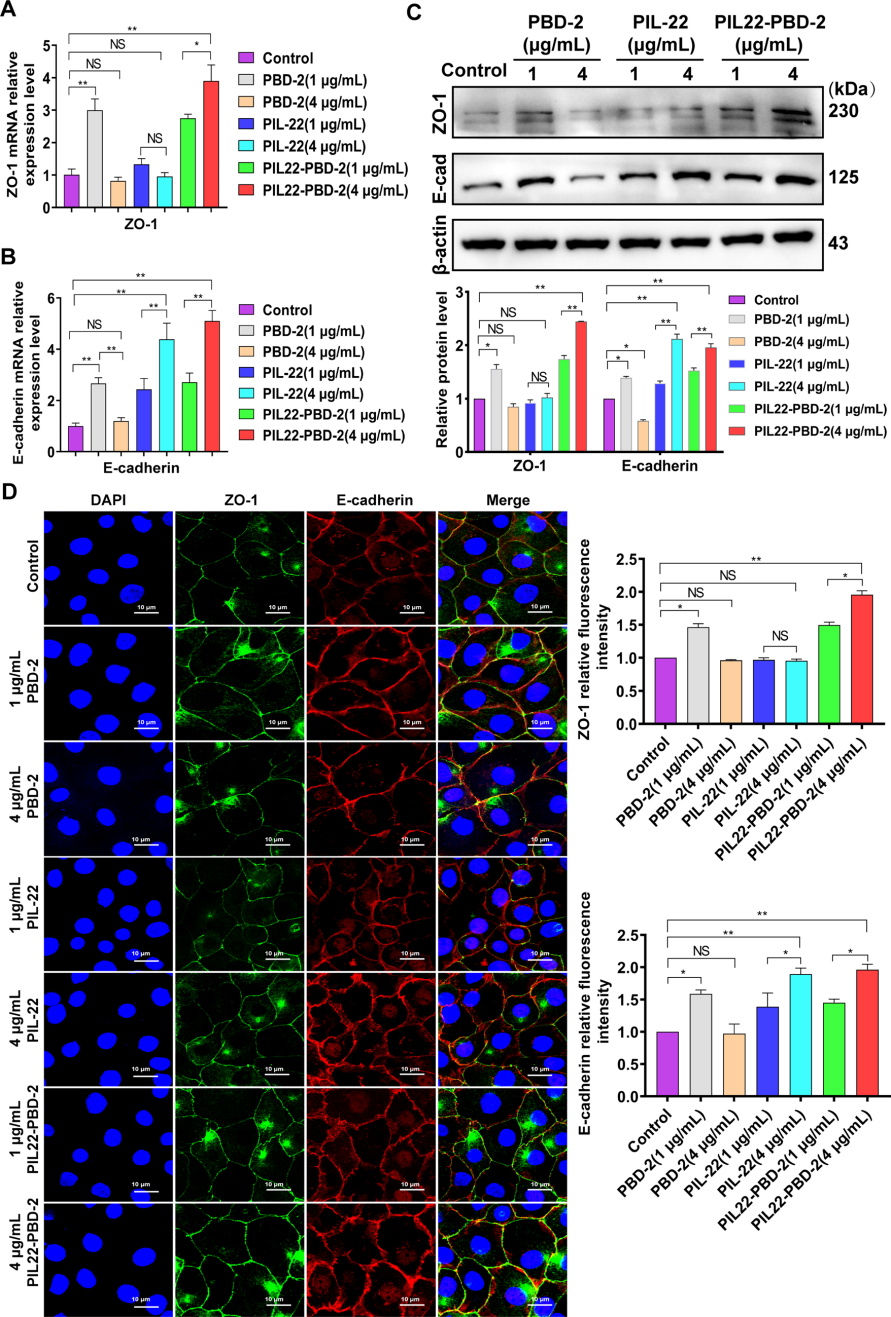
**The PIL22-PBD-2 promotes the expression of intercellular junction proteins of IPEC-J2.** qPCR analysis for ZO-1 (**A**) and E-cadherin (**B**). **C** Western blot analysis for intercellular junction protein ZO-1 and E-cadherin. **D** Immunofluorescence analysis for ZO-1 and E-cadherin. PBS treatment was used as control. Error bars indicate standard deviations. *p < 0.05 and **p < 0.01 were compared by two-way ANOVA followed by Bonferroni’s multiple comparisons test.

To determine whether three proteins promote the expression of intercellular junction proteins (ZO-1 and E-cadherin) in IPEC-J2, we performed qRT-PCR and Western blot assays to examine mRNA abundances and protein levels of ZO-1 and E-cadherin in IPEC-J2. Our data showed that PIL-22 treatment substantially induced E-cadherin expression in a concentration-dependent manner (Figures 4B and C), while it had no significant effect on ZO-1 expression (Figures 4A and C). However, both ZO-1 and E-cadherin expression increased in the PIL22-PBD-2 treatment group in a concentration-dependent manner (Figures 4A–C). Compared to the control group, the concentration of PBD-2 at 4 μg/mL did not impact the increase of intercellular junction proteins. However, at a concentration of 1 μg/mL, PBD-2 enhanced the expression of ZO-1 and E-cadherin compared to the control group, as shown in Figures 4A–C.

To further understand the relationship between intercellular junction proteins and three proteins, we examined the cellular distribution of ZO-1 and E-cadherin in IPEC-J2 cells after treating them with proteins (Figure 4D). The immunofluorescence microscopy results showed a pronounced distribution of intercellular junction proteins at the cell–cell junction in PIL22-PBD-2-treated IPEC-J2 cells compared to the control group. However, in PIL-22-treated cells, there was no significant change in ZO-1 staining compared to the control group. However, the distribution of E-cadherin at the cell–cell junction increased. Additionally, low concentrations of PBD-2-treated cells significantly increased the distribution of intercellular junction proteins compared to the control group.

In contrast, treating cells with high concentrations of PBD-2 did not considerably alter the localisation of intercellular junction proteins at cell junctions. We hypothesise that the cellular damage caused by high concentrations of PBD-2 may impact the distribution of connexin proteins. These findings suggest that the recombinant antibacterial protein PIL22-PBD-2 exhibits characteristics of both PBD-2 and PIL-22.

### The PIL22-PBD-2 alleviates the effect of ETEC O8 infection with IPEC-J2

**Figure 5:**
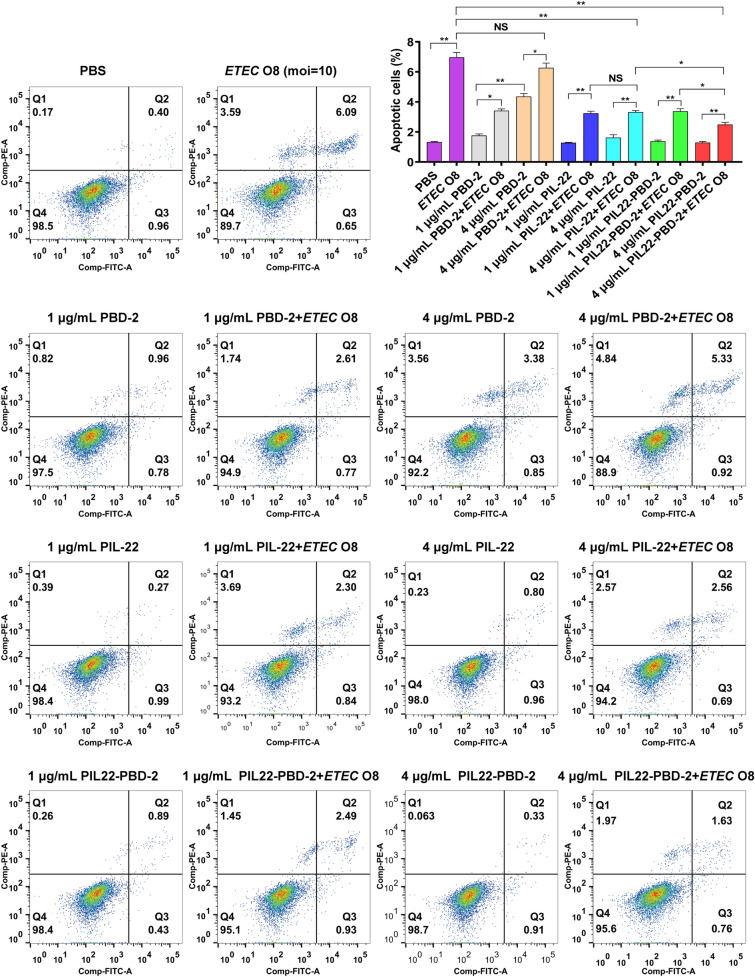
**The effect of PIL22-PBD-2 on apoptosis in ETEC O8-infected cells was detected using flow cytometry.** Frames were divided into four quadrants: Q1 represents necrotic cells; Q2 represents late-stage apoptotic cells; Q3 represents early-stage apoptotic cells; Q4 represents normal cells; Control, sterile PBS; and error bars indicate standard deviations. *p < 0.05 and **p < 0.01 were compared by two-way ANOVA followed by Bonferroni’s multiple comparisons test.

To evaluate the protective effect of PIL22-PBD-2 against ETEC O8 infection, we examined apoptosis, bacterial adhesion, mRNA levels of endogenous defensin, and inflammatory cytokines in IPEC-J2 cells infected with ETEC O8. As shown in Figure 5, when IPEC-J2 cells were exposed to the ETEC O8 challenge and pre-treated with 4 μg/mL PBD-2, there was an increase in apoptosis and necrotic rates compared to the PBS group (*p* < 0.01). Moreover, compared with the ETEC O8 challenge group, pretreatment with 4 μg/mL of PIL-22 or PIL22-PBD-2 significantly decreased apoptosis and necrosis rates (*p* < 0.01).

**Figure 6:**
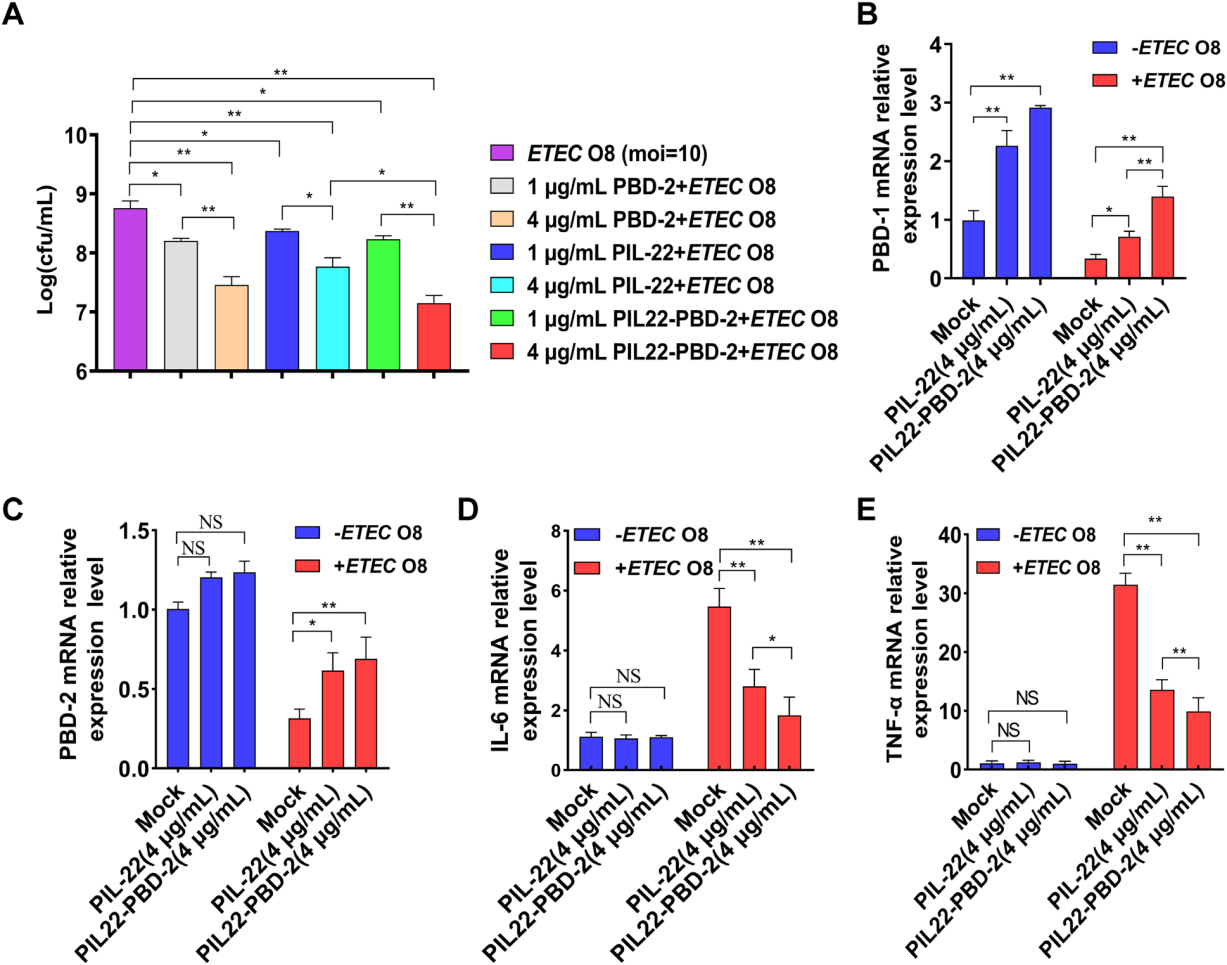
**The PIL22-PBD-2 alleviates the effect of ETEC O8 infection on IPEC-J2. A** PIL22-PBD-2 decreased ETEC O8 adhesion. Cells pre-treated with PIL22-PBD-2, PIL-22 and PBD-2 were incubated with equal numbers of ETEC O8 for 4 h, and the number of adherent bacteria (CFU) was determined. Real-time PCR analysis of PBD-1 (**B**) and (**C**) PBD-2 mRNA abundance in IPEC-J2 infected by ETEC O8. Real-time PCR analysis of IL-6 (**D**) and TNF-α (**E**) mRNA abundance in IPEC-J2 infected by ETEC O8. Error bars indicate standard deviations. *p < 0.05 and **p < 0.01 were compared by two-way ANOVA followed by Bonferroni’s multiple comparisons test.

Notably, PIL22-PBD-2 treatment significantly reduced the percentage of apoptotic cells compared to PIL-22 treatment at the same concentration of 4 μg/mL. Moreover, PBD-2, PIL-22, and PIL22-PBD-2 effectively reduced the adhesion of ETEC O8 compared to the ETEC O8 challenge control groups in a concentration-dependent manner (Figure 6A). A greater inhibitory effect on the adhesion of ETEC O8 was detected in PIL22-PBD-2-treated cells compared to PIL22-treated cells at the same concentration (*p* < 0.05). Since PIL-22 has been shown to play a role in inducing PBD endogenous expression [8], we detected endogenous beta-defensin expression in the cells treated with PIL-22 or PIL22-PBD-2, with or without the presence of ETEC O8.

Our data showed that PIL22-PBD-2 treatment stimulated more porcine beta-defensin 1 (PBD-1) expression than PIL22 treatment, whether in the presence or absence of ETEC O8 (Figure 6B). Furthermore, no significant difference was detected in the expression of PBD-2 in the cells treated with PIL-22 or PIL22-PBD-2 compared to untreated cells (Figure 6C). The ETEC O8 challenge significantly elevated the expression levels of inflammatory cytokines such as IL-6 and TNF-α [44]. Our results also showed that PIL-22 and PIL22-PBD-2 treatments substantially decreased IL-6 (Figure 6D) and TNF-α (Figure 6E) mRNA expression in cells exposed to ETEC O8 (*p* < 0.01). Treatment with PIL22-PBD-2 had a greater inhibitory effect on TNF-α mRNA expression than PIL-22 treatment (*p* < 0.05).

In conclusion, these results establish that the PIL22-PBD-2 treatment attenuated the ETEC O8-induced inflammatory responses in IPEC-J2 cells by exerting antibacterial activity, thus increasing the expression of endogenous AMPs and significantly decreasing the mRNA expression levels of IL-6 and TNF-α (*p* < 0.05).

## Discussion

Due to dual factors, the increasingly severe microbial resistance to antibiotics and the policy of replacing and reducing antibiotics, there is an urgent need to develop antibiotic substitutes as feed additives in animal husbandry [13]. AMPs, especially defensins, are highly effective natural antibacterial agents that play a vital role in the host’s body by forming the first line of defence against pathogens [45–47]. PBD-2 is renowned for its early discovery, extensive research, broad-spectrum antibacterial activity against pathogenic intestinal bacteria, and excellent immunomodulatory effects in pigs [23, 48]. Furthermore, IL-22 is a cytokine found to inhibit bacterial adhesion, protect against enteric coronavirus infection [49], and promote the repair of intestinal damage [8, 31]. Interestingly, PIL-22 can induce IPEC-J2 cells to produce defensins that fight bacterial infections [8].

Post-weaning stress in piglets can easily result in pathogenic bacteria infecting the intestinal tract. Such infections can cause severe damage to intestinal villi, increase inflammatory factors, decrease tight junction protein expression, and damage the growth performance of piglets [50–52]. PBD-2 has shown rapid, broad-spectrum bacteriostatic properties, while IL-22 has a strong and long-lasting ability to repair the intestine. These properties make both compounds promising alternatives to antibiotics for preventing and treating intestinal bacterial infections in piglets. Here, we efficiently expressed the antibacterial protein PIL22-PBD-2 by *P. pastoris* and demonstrated that PIL22-PBD-2 has potential as a new feed additive.

Selecting a suitable linker is essential for joining the protein domains in recombinant fusion proteins. It can help avoid undesirable outcomes, including misfolding of the fusion proteins, low protein production yield, or impaired bioactivity [39]. The (GGGGS)n is the most commonly used flexible linker with a sequence consisting primarily of stretches of Gly and Ser residues. By adjusting the copy number “n”, the length of this GS linker can be optimised to achieve appropriate separation of the functional domains or to maintain necessary inter-domain interactions [39]. For this study, we selected the flexible linker (GGGGS)_2_ to connect two functional proteins, ensuring their biological function remained unaffected [53]. Previous studies have shown that disulfide bonds in peptides play a critical role in their antibacterial activity [4]. However, antibacterial peptides can only achieve their best antibacterial effect when they are in a specific structural state [54]. Therefore, we hypothesised that to ensure better antibacterial activity, the intramolecular disulfide bond pattern of *P. pastoris* expressed PIL22-PBD-2 must match that of natural PBD-2 [4, 55].

Compared to traditional antibiotics, AMPs have an advantage in killing resistant bacteria because their mechanism of destroying the bacterial membrane makes it difficult for bacteria to develop drug resistance [9]. The MIC and Oxford Cup tests showed that PIL22-PBD-2 had good antibacterial activity against gram-negative and gram-positive bacteria. More importantly, compared to antibiotics, PIL22-PBD-2 had a non-specific bactericidal effect on the multidrug-resistant ETEC O8 isolated from piglets with clinical diarrhoea. The impact of PIL22-PBD-2 on the ultrastructure of *E. coli*, *Salmonella*, and *S. aureus* was visualised using TEM. Cell rupture, vacuole formation, and plasma membrane contraction were observed at the MIC, consistent with previous studies on PBD-2 [48].

Research has shown that the drug dosage is a central factor affecting the drug’s effectiveness. Varying doses of the same drug may result in fluctuating effects, and excessive doses may lead to adverse reactions [56]. Similarly, the CCK8 results indicated that the viability of IPEC-J2 cells was affected differently by various concentrations of three proteins. PIL-22 and PIL22-PBD-2 at concentrations ranging from 1 μg/mL to 4 μg/mL significantly enhanced cell survival rates, suggesting that optimal protein concentrations can stimulate the growth of intestinal epithelial cells. However, a concentration of 4 μg/mL of PBD-2 showed cytotoxic effects on the cells. Wound healing experiments demonstrated that PIL22-PBD-2 and PIL-22 both promote cell scratch healing at an appropriate concentration range. Some studies reported similar results, indicating that PIL-22 could regulate epithelial proliferation and differentiation after cell injury [31]. Although studies have also shown that PBD-2 has a certain ability to promote cell proliferation [7, 56], the effect of PBD-2 at a concentration of 1 μg/mL on cell proliferation is not significant. Therefore, we believe that the optimal dosage of PBD-2 needed to carry out its biological functions may need to be reevaluated in light of its strong cationic activity.

Intercellular junctions, including tight junctions and adherens junctions, play an essential role in maintaining the integrity of the intestinal barrier and resisting the invasion of harmful substances and microbiota [57, 58]. ZO-1 is an important component of tight junctions and the related cytoskeleton, playing a vital role in maintaining cell permeability [58, 59]. E-cadherin is a classical adherens junction protein that is mainly responsible for initiating and maintaining cell–cell adhesion and regulating the organisation of the underlying actin cytoskeleton [58]. Given their central role in the gut, we sought to clarify the effects of PIL22-PBD-2 on the expressions of ZO-1 and E-cadherin in IPEC-J2. It has been demonstrated that PBD-2 can promote the expression of ZO-1 protein in a DSS-treated mouse model [7], while PIL-22 can only promote the expression of E-cadherin and has no effect on ZO-1 [8].

Our results indicate that PBD-2, at a concentration of 1 μg/mL, increased the mRNA and protein levels of ZO-1 and E-cadherin compared to the control group (*p* < 0.01). PIL22-PBD-2 also significantly promotes both ZO-1 and E-cadherin expression in IPEC-J2. This finding suggests that positive alterations to both tight and adherens junctions occurring in the cell enhance the integrity of the intestinal barrier, potentially improving the ability to resist invasion by intestinal bacteria [59].

Furthermore, as a form of cell death in physiology, apoptosis plays an important role in regenerating intestinal mucosal cells. In cases of severe intestinal disease, the intestinal mucosa can break down more quickly than usual, leading to accelerated apoptosis and increased intestinal permeability [7]. Bacteria such as ETEC O8 can affect the expression of tight junction proteins, increase intestinal permeability, damage intestinal mucosa, and cause intestinal inflammation and diarrhoea [9, 60, 61]. Previous studies have demonstrated that IL-22 is primarily responsible for regulating the expression of AMPs, activating the STAT3 signalling pathway, which is related to inhibiting apoptosis, maintaining the function of the intestinal epithelial barrier, improving intestinal inflammation, and ultimately preventing gastrointestinal bacterial infections [62]. In the present study, the ETEC O8 challenge significantly increased apoptosis and necrosis rates in IPEC-J2. However, pretreatment with PIL22-PBD-2 reduced the percentage of apoptotic and necrotic cells in ETEC O8-challenged IPEC-J2.

Moreover, treatment with PBD-2 and PIL-22 of 1 μg/mL concentration has been shown to partially resist ETEC O8-induced cell apoptosis, which may be related to their antibacterial abilities and increased expression of intercellular junction proteins. To further understand the mechanisms behind how PIL22-PBD-2 modulates intestinal barrier functions, we explored the expression levels of some key molecules involved in the inflammatory response. Interestingly, PIL22-PBD-2 and PIL-22 treatments substantially decrease the expression levels of several critical inflammatory cytokines (i.e., IL-6 and TNF-α) in ETEC O8-challenged cells compared to untreated cells. We found that the adhesion of ETEC O8 in the PIL22-PBD-2 treatment group was considerably lower than in the ETEC O8 control group (*p* < 0.01), which may be related to its antibacterial activity and increased expression of endogenous defensins. The positively charged peptide can neutralise the negatively charged endotoxin, thus reducing the body’s inflammatory response [9]. Further studies are needed to address the effects of PIL22-PBD-2 on anti-apoptotic and anti-infective activity in vivo.

Numerous studies have demonstrated that the production of endogenous defensins plays a crucial role in the body’s ability to resist bacterial or viral infections [47, 63]. Despite the low concentration of defensins in physiological states, this does not prevent defensins from mounting an effective defence mechanism in vivo. This response is two-fold as their expression is highly localised, and their presence is at high concentrations when in action [47]. In addition, endogenous defensin is involved in regenerating barrier cells against bacterial infection [63]. Our study also demonstrated that the fusion antibacterial protein PIL22-PBD-2 not only acts directly during bacterial infection but also induces the production of endogenous defensin (PBD-1) and promotes the integrity of IPEC-J2 in vitro.

Furthermore, we found that PIL-22 and PIL22-PBD-2 did not increase the expression of PBD-2 mRNA at the concentrations tested. This outcome was consistent with the findings of previous studies [8]. Despite our results showing that PIL22-PBD-2 has excellent biological activity in vitro, as a foreign protein, it is easily hydrolysed and degraded by a variety of proteases, polysaccharides, bivalent cations, and nucleic acids in vivo, resulting in poor bioavailability and efficacy [64]. In the future, we will focus on loading PIL22-PBD-2 onto delivery systems that are rationally designed. Such an approach will help maintain its stability and activity, alleviate its susceptibility to proteolytic degradation, and evaluate its application in animals.

In summary, this study is the first to reveal that the recombinant protein of PIL22-PBD-2 has a positive effect on inhibiting pathogenic bacteria and repairing intestinal damage. These results will provide a valuable reference for further studies on AMPs as a clinical alternative to antibiotics.

## Supplementary Information

**Additional file 1.** **Expression of PBD-2 and PIL-22 proteins in** ***P. pastoris***. (A) Colony PCR identification of recombinant P. pastoris containing PBD-2 gene. Lane M: DL2000 DNA marker; Lane 1: negative control; Lane 2 and 3: a 243 bp fragment was amplified with PBD-2 gene primers; Lane 4 and 5: a 724 bp fragment was amplified with P. pastoris AOX1 primers; SDS-PAGE (B) and WB (C) analyses of PBD-2 protein. Lane M: protein marker (11-245 kDa). Lane 1: the fermentation supernatant of blank vector as the negative control. Lane 2: the fermentation supernatant of PBD-2; Lane 3: purified PBD-2 protein. (D) Colony PCR identification of recombinant P. pastoris containing PIL-22 gene. Lane M: DL2000 DNA marker; Lane 1: negative control; Lane 2: The p9K vector was electrotransfected into yeast, then amplified with AOX1 primer to generate a 481 bp fragment; Lane 3-5: a 1003 bp fragment and a 522 bp fragment were amplified with P. Pastoris AOX1 primers and PIL-22 gene primers, respectively; SDS-PAGE (E) and WB (F) analyses of PIL-22 protein. Lane M: protein marker (11-245 kDa). Lane 1: the fermentation supernatant of blank vector as the negative control. Lane 2: the fermentation supernatant of PIL-22; Lane 3: purified PIL-22 protein.

**Additional file 2.  ETEC O8 serotype identification and drug sensitivity testing**. (A) ETEC O8 serotype identification. Lane M: DL2000 DNA marker; Lane 1: negative control; Lane 2 and 3: The 448 bp fragment was amplified with ETEC O8 specific gene orf469 primer. (B) Representative results of drug susceptibility testing for ETEC O8 strain. 1: Kanamycin (30 μg); 2: Ampicillin (10 μg); 3: Streptomycin (10 μg); 4: Cefpiramide (30 μg); 5: Tobramycin (10 μg); 6: Ofloxacin (5 μg); 7: PBS.

**Additional file 3.** **Optimised nucleotide sequences of the PIL22-PBD-2.**

## Data Availability

All data supporting the findings of this study are available within the article or from the corresponding author upon reasonable request.
