# Supplementary figures and images for: Expression of recombination antimicrobial protein PIL22-PBD-2 in Pichia pastoris and verification of its biological function in vitro
Source: Vet Res. 2025 Mar 7;56:52. doi: 10.1186/s13567-024-01428-1 (PMC11889930; doi:10.1186/s13567-024-01428-1)

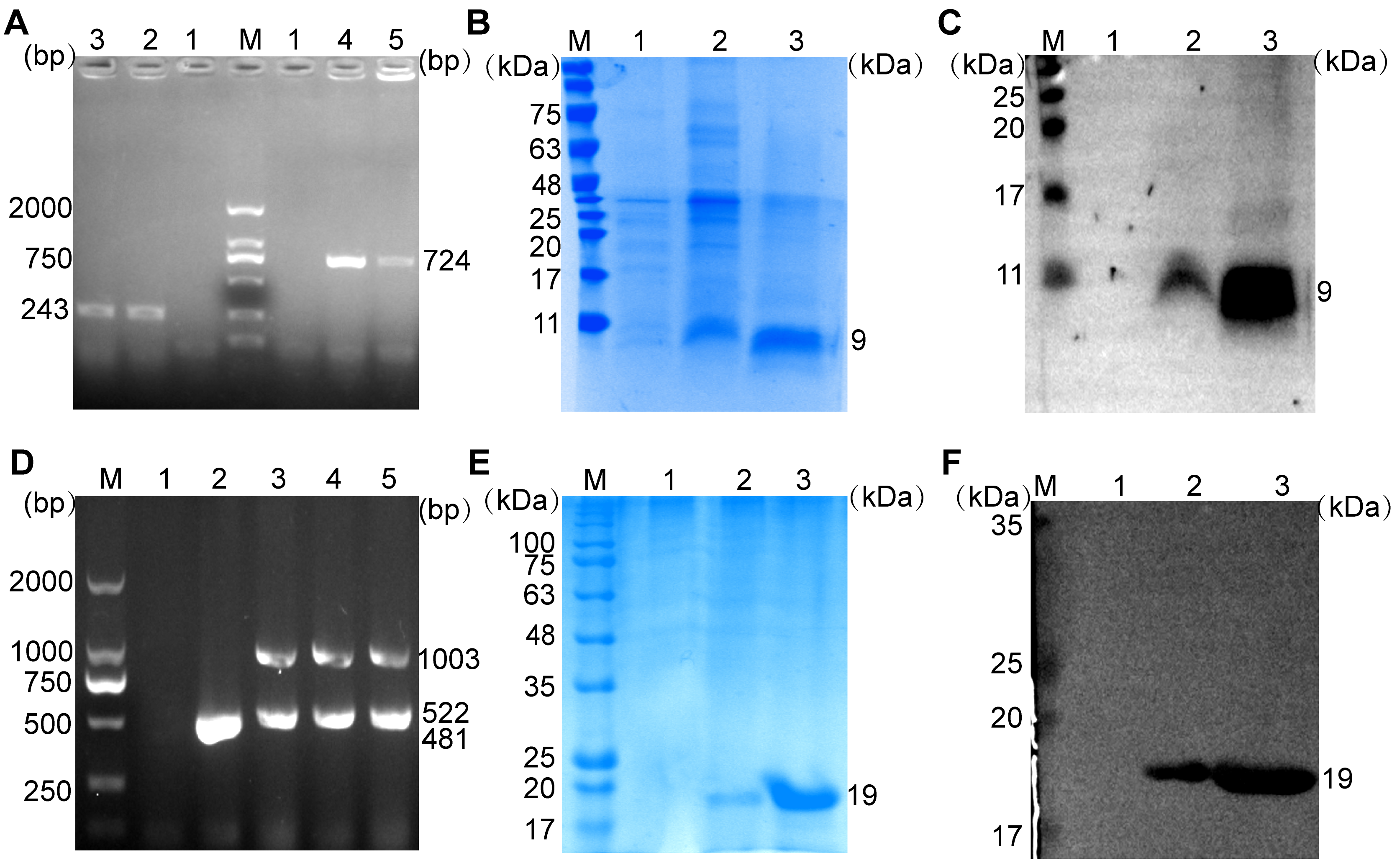

Supplement: Supplementary file 1 — Additional file 1. Expression of PBD-2 and PIL-22 proteins in P. pastoris. (A) Colony PCR identification of recombinant P. pastoris containing PBD-2 gene. Lane M: DL2000 DNA marker; Lane 1: negative control; Lane 2 and 3: a 243 bp fragment was amplified with PBD-2 gene primers; Lane 4 and 5: a 724 bp fragment was amplified with P. pastoris AOX1 primers; SDS-PAGE (B) and WB (C) analyses of PBD-2 protein. Lane M: protein marker (11-245 kDa). Lane 1: the fermentation supernatant of blank vector as the negative control. Lane 2: the fermentation supernatant of PBD-2; Lane 3: purified PBD-2 protein. (D) Colony PCR identification of recombinant P. pastoris containing PIL-22 gene. Lane M: DL2000 DNA marker; Lane 1: negative control; Lane 2: The p9K vector was electrotransfected into yeast, then amplified with AOX1 primer to generate a 481 bp fragment; Lane 3-5: a 1003 bp fragment and a 522 bp fragment were amplified with P. Pastoris AOX1 primers and PIL-22 gene primers, respectively; SDS-PAGE (E) and WB (F) analyses of PIL-22 protein. Lane M: protein marker (11-245 kDa). Lane 1: the fermentation supernatant of blank vector as the negative control. Lane 2: the fermentation supernatant of PIL-22; Lane 3: purified PIL-22 protein. [file 13567_2024_1428_MOESM1_ESM.tif]

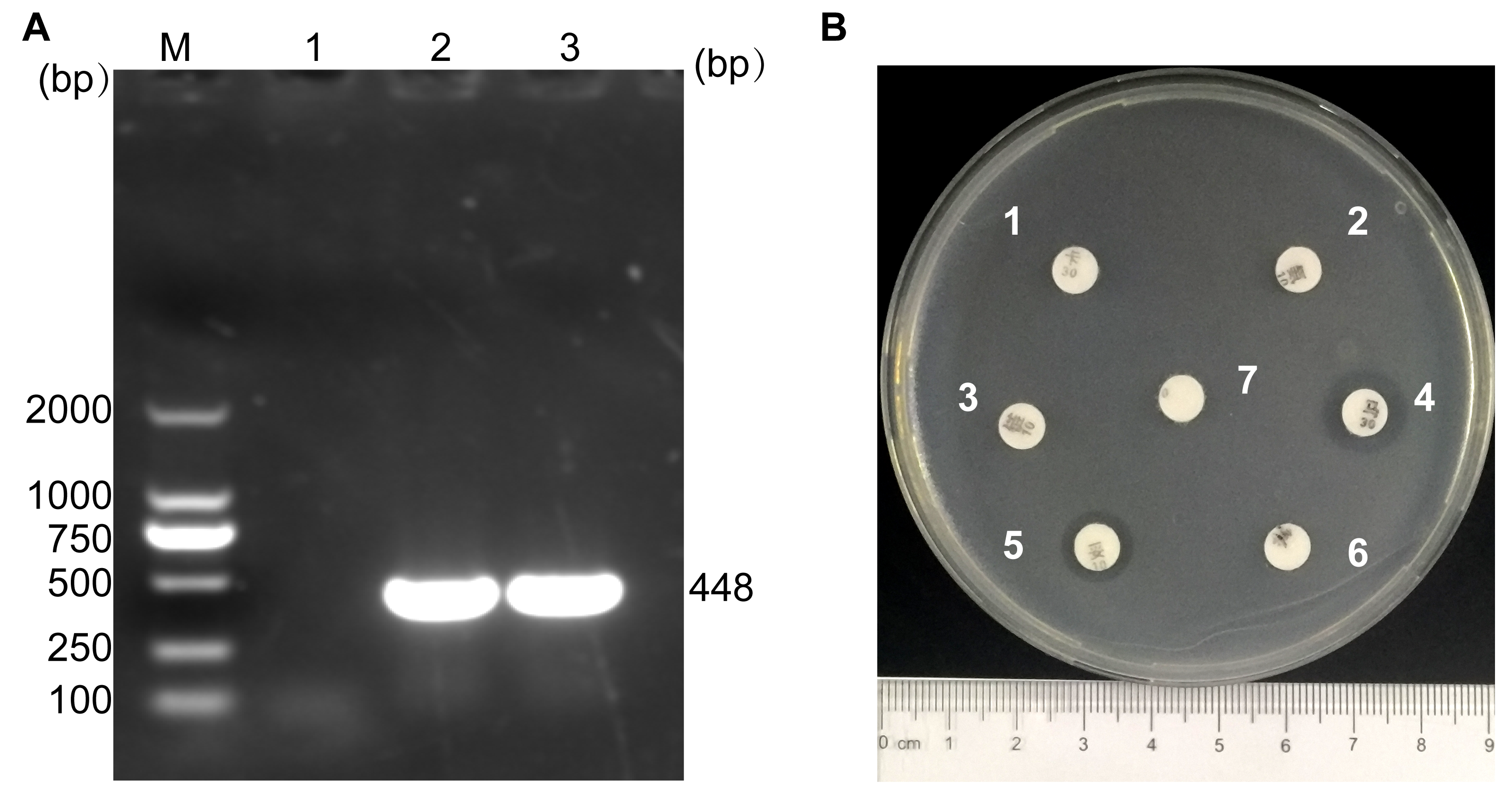

Supplement: Supplementary file 2 — Additional file 2. ETEC O8 serotype identification and drug sensitivity testing. (A) ETEC O8 serotype identification. Lane M: DL2000 DNA marker; Lane 1: negative control; Lane 2 and 3: The 448 bp fragment was amplified with ETEC O8 specific gene orf469 primer. (B) Representative results of drug susceptibility testing for ETEC O8 strain. 1: Kanamycin (30 μg); 2: Ampicillin (10 μg); 3: Streptomycin (10 μg); 4: Cefpiramide (30 μg); 5: Tobramycin (10 μg); 6: Ofloxacin (5 μg); 7: PBS. [file 13567_2024_1428_MOESM2_ESM.tif]
