# Supplementary material for: Expression of recombination antimicrobial protein PIL22-PBD-2 in Pichia pastoris and verification of its biological function in vitro
Source: Vet Res. 2025 Mar 7;56:52. doi: 10.1186/s13567-024-01428-1 (PMC11889930; doi:10.1186/s13567-024-01428-1)
Supplement: Supplementary file 3 — Additional file 3. Optimised nucleotide sequences of the PIL22-PBD-2. [file 13567_2024_1428_MOESM3_ESM.docx]

**Additional file 3** **Optimized nucleotide sequences of the PIL22-PBD-2.**

| Name | Nucleotide Sequence |
| --- | --- |
| PIL22-PBD-2 | 5′ATGCCCATTACTCATCATTGCAAACTCGACCAGAGCAACTTCCAGCAACCTTACATAACGAATAGGACGTTTACACTGGCGCAGGAGGCGTCTCTAGCTGACAATAATACGGACGTACGGCTTATTGGGAATAACCTTTTCCAAGGCGTTAATCAAATGCGAGAGAGGTGTTATCTGGTCAAGCAAGTCCTCAATTTTACTTTGGAGGAAGTATTATTTCCCAATAGTGATCGGTTTCATCCATATATGCAGGAAGTGGCCTCCTTCCTCGACAGTCTTTCGAAAAAACTATCCCAATGTAGAATTAAGGGTGACGATCAACACATACAGCGAAACGTGAACAATTTCAAGGATATCGTTAAGAAGCTGGGAGAATCTGGCGAAATCAAAGTTATAGGGGAGTTGTACCTACTCTTCATGGCTCTAAAGAACGAATGTACCCTACCGGGACACAGTTGGAAAATGGATAATGGAGGTGGGGGATCAGGGGGCGGGGGGTCCCGCGCCCTGTGTCTGTTATTATTAACCGTGTGCTTGCTCTCGTCACAGTTGGCAGCCGGCATAAACCTTTTGACCGGACTTGGGCAGAGATCGGATCACTATATTTGCGCGAAGAAAGGCGGAACATGTAACTTCTCTCCGTGCCCATTATTTAACCGTATCGAGGGAACTTGTTACTCAGGCAAGGCAAAATGCTGCATCCGTGGCGGTGGAGGGAGCCACCACCATCATCACCACTGA-3′ |
